# Mitochondrial DNA nucleotide changes in primary congenital glaucoma patients

**Published:** 2013-02-01

**Authors:** Manoj Kumar, Mukesh Tanwar, Muneeb Ahmad Faiq, Jhumur Pani, Monis Bilal Shamsi, Tanuj Dada, Rima Dada

**Affiliations:** 1 Laboratory for Molecular Reproduction and Genetics, Department of Anatomy, All India Institute of Medical Sciences, New Delhi, India; 2 Dr. Rajendra Prasad Centre for Ophthalmic Sciences, All India Institute of Medical Sciences, New Delhi, India

## Abstract

**Purpose:**

Primary congenital glaucoma (PCG) is the second most common cause of blindness, accounting for 0.01%–0.04% of total blindness worldwide. Most congenital glaucoma cases are mapped to the GLC3A locus, and many aspects of PCG are still unknown. Recent studies have reported an increased frequency of mitochondrial DNA (mtDNA) sequence changes in primary open-angle glaucoma, primary angle-closure glaucoma, and pseudoexfoliation glaucoma compared to controls. Thus, this study was planned with the aim of detecting mitochondrial DNA variations in PCG cases.

**Methods:**

Twenty primary congenital glaucoma cases were selected from Dr. R. P. Centre for Ophthalmic Sciences of All India Institute of Medical Sciences (AIIMS), New Delhi, India. DNA was isolated from whole blood samples. The entire coding region of the mitochondrial genome was amplified by PCR in 20 patients and 20 controls. The full mtDNA genome was sequenced and analyzed against mitochondrial reference sequence NC_012920.

**Results:**

MtDNA sequencing revealed a total of 195 nucleotide variations in PCG patients and 58 in controls. Of the 195 changes, 43 (22.05%) were nonsynonymous, 82 (42.05%) were synonymous, and 30 were in RNA genes. A total of 39/195 (20.00%) variations were observed in the D-loop (hypervariable region), 19/195 (9.74%) in different ribosomal RNA (rRNAs), 11/195 (5.64%) in transfer RNA (tRNAs), 66/195 (33.84%) in complex I, 17/195 (8.71%) in complex III, 27/195 (13.84%) in complex IV, and 15/195 (7.69%) in complex V. Of 58 variations in the controls, 14 were nonsynonymous changes. The Sorting Intolerant from Tolerant and Polymorphism Phenotyping analyses of all nonsynonymous changes from patients revealed two pathogenic changes in NADH-ubiquinone oxidoreductase chain 2 (ND2) and cytochrome oxidase subunit III (COXIII) subunits. In one of the patients, the insertion of cytosine introduced a frame shift change (p.Ile104AsnfsX26) in the cytochrome b (CYB) subunit of the electron transport chain. In another patient, a variation (G8572A) in ATP synthase 8 (ATpase8) led to the introduction of a stop codon or termination at amino acid position 69. Haplogroup/phylogenetic analysis of mtDNA showed that primary congenital glaucoma patients belong to three macrohaplogroups: M (4), N (15), and L (1). Fifty percent of the patients belonged to the H2a2a lineage of the N-derived haplogroup.

**Conclusions:**

Although several mutations were found at a higher frequency among our population, there is a need to complement this study with functional studies and to analyze a large number of samples in different populations of different haplogroups, as penetrance varies among haplogroups.

## Introduction

Glaucoma is a heterogeneous group of eye conditions with manifestation as early as birth to very late age of onset. Primary congenital glaucoma (PCG) is the second most common cause of blindness, accounting for 0.01%–0.04% of total blindness worldwide. PCG; OMIM 231300; provided in the public domain by the National Centre for Biotechnology Information, Bethesda, MD) is a severe form of glaucoma with manifestation at birth or early childhood. It is characterized by elevated intraocular pressure (IOP), and enlarged cornea and globe (buphthalmos) [1]. PCG refers to a specific form of developmental glaucoma characterized by an isolated trabeculodysgenesis (which leads to impaired aqueous drainage, increased IOP, and optic nerve damage, and may ultimately lead to partial/permanent visual impairment) that is not associated with other developmental ocular anomalies or ocular disease that can raise the IOP. Also called primary infantile glaucoma, it is the most common form of developmental glaucoma. The condition is typically bilateral, but 25%–30% of cases may be unilateral.

Most PCG cases present within the first year of life, out of which 25% are diagnosed in the neonatal period, and about 60% within the first six months of life. The majority of PCG cases are sporadic. PCG is bilateral in 80% of cases; it is the most common type of pediatric glaucoma, accounting for 55% of such glaucoma. The incidence of PCG is different in different populations. The prevalence of PCG varies across ethnic communities, ranging from 1 in 10,000–20,000 in the western Western populations [2] to 1 in 2,500 Saudi Arabian population [3], 1 in 1,250 in the Gypsy population of Slovakia [2] and 1 in 3,300 in Andhra Pradesh, India [4]. The majority of patients (about 60%) are diagnosed by the age of six months, and 80% are diagnosed within the first year of life. A slight predominance of males is common (about 65%), and involvement is usually bilateral (about 70%).

The majority of congenital glaucoma maps to the cytochrome P450, family 1, subfamily B, polypeptide 1 (GLC3A) locus on chromosome 2 (2p21). Three genetic loci—GLC3A (2p21), GLC3B (1p36), and GLC3C (14q24.3-q31.1)—have been mapped by linkage analysis [3,5,6]. Mutations in the *CYP1B1* gene in the GLC3A locus are associated with the PCG phenotype. This is the predominant genetic pattern of PCG in the Middle East (Turkey and Saudi Arabia). It has been reported that 87% of familial and 27% of sporadic cases are due to mutations in this gene [3-11]. Digenic inheritance is an inheritance mechanism resulting from the interaction of two nonhomologous genes; in glaucoma, digenic inheritance has been shown recently in two instances, specifically early-onset glaucoma in humans and in mice. Cytochrome P450, family 1, subfamily B, polypeptide 1 (*CYP1B1*) and myocilin, trabecular meshwork inducible glucocorticoid response (*MYOC*) mutations were identified in early-onset glaucoma in humans, whereas mutations in the *CYP1B1* and *FOXC1* were detected in mice with early-onset glaucoma. It is estimated that all known loci/genes of glaucoma account for the minority of total cases of glaucoma, and thus, many other genes remain to be identified. Many aspects of PCG are still unknown and current treatment options are not sufficient to block neurodegenerative injury in these cases. Early and reliable diagnosis of this disease is vital so that appropriate and prompt treatment can be initiated. This can improve outcomes for vision and prevent visual loss.

The role of mitochondrial DNA (mtDNA) mutations and oxidative stress (OS) has been reported in primary open-angle glaucoma (POAG) [9,12]. Recent studies reported an increased frequency of mtDNA sequence changes in POAG, primary angle-closure glaucoma (PACG), and pseudoexfoliation glaucoma (PEG) compared to controls [9,13,14]. This study was planned with the aim of detecting mitochondrial DNA variations in PCG cases. In a previous study from our laboratory, we reported the role of mtDNA variations in PCG cases and postulated how mtDNA sequence variants which lead to mitochondrial dysfunction and thus depleted ATP levels result in trabecular dysgenesis and retinal ganglion cell (RGC) death. As we detected a large number of sequence variations in a previous study [15], we planned to study if there were any recurrent mutations in PCG cases. Thus, in this study, we analyzed complete mitochondrial genomes in 20 cases of PCG that were negative for mutations in the CYP1B1, MYOC, FOXC1, and LTBP2 genes [16].

## Methods

### Clinical examination and selection of cases

Twenty cases of PCG that were negative for mutations in the CYP1B1, MYOC, FOXC1, and LTBP2 genes [16] and presenting at the Dr. R. P. Centre for Ophthalmic Sciences (All India Institute of Medical Sciences [AIIMS], New Delhi, India) were included for this study, after ethical approval of the Institutional Review Board (IRB00006862; All India Institute of Medical Sciences, New Delhi, India). The diagnosis involved clinical ocular and systemic examination. Inclusion criteria of the patients were as follows: increased corneal diameter (>12.0 mm), raised IOP (>21 mmHg) with the presence/absence of Haab’s striae, and optic disc changes (where examination was possible). Symptoms of epiphora and photophobia were additional inclusion factors. All patients with a history of blood transfusion, toxoplasmosis/rubella/cytomegalovirus/herpes simplex virus (TORCH) infection, and drug intake in the mother during pregnancy were excluded. Glaucoma cases other than PCG were also excluded. Detailed family history of ocular or other hereditary disorders up to three generations were taken, and pedigree charts were constructed. Twenty ethnically matched normal individuals without any ocular disorders with IOP<20 mmHg and corneal diameter <12×12 mm were enrolled as controls. Sample collection and DNA isolation: Peripheral blood samples were collected from patients and controls by venipuncture after informed consent. Blood samples were collected in EDTA vaccutainers and stored in **-**80 °C until DNA isolation. DNA was isolated from whole blood using the phenol-chloroform method.

### Molecular analysis

The whole mitochondrial genome was amplified in all patients and controls using 24 pairs of primers. PCR amplifications for all primer sets were performed in a 40 μl volume containing 1.0 μl of 20 μM stock solution for each primer, 100 ng of genomic DNA, 1 unit of Taq polymerase (Banglore Genei, Bengaluru, Karnataka, India), 0.1 mM of each dNTP, 4 μl of 10×PCR buffer (with 15 mM MgCl_2_), by means of 30 cycles of amplification, each consisting of 30 s denaturation at 94 °C, 30 s annealing at 56 °C, and 1 min extension at 72 °C. Finally, an extension for 5 min at 72 °C was performed. Amplified PCR products were purified using a gel/PCR DNA fragments extraction kit (catalog number DF100; GeneaidBiotech Ltd., Sijhih City, Taiwan). Purified PCR products were sent for sequencing to Molecular Cloning Laboratories (South San Francisco, CA). All fragments were sequenced in both forward and reverse direction for confirmation. All sequence variants from both PCG patients and controls were compared to the human mitochondrial reference sequence NC_012920 provided by the National Center for Biotechnology Information (NCBI) using ClustalW2 (a multiple sequence alignment program for DNA; European Molecular Biology Laboratory–European Bioinformatics Institute). The mtDNA haplogroup was constructed to check whether any specific haplogroup was associated with PCG.

### Prediction of pathogenicity

For the prediction of the pathogenic characteristics of all nonsynonymous mtDNA changes, two homology-based programs—the Polymorphism Phenotyping (Polymorphism Phenotyping) and Sorting Intolerant from Tolerant (SIFT) analysis tools—were used. PolyPhen structurally analyzes an amino acid polymorphism and predicts whether that amino acid change is likely to be deleterious to protein function. The prediction is based on the position-specific independent counts score derived from multiple sequence alignments of observations in the functional domain of a protein, or predicted hydrophobic and transmembrane matrix element difference in the transmembrane region of a protein. PolyPhen scores of >2.0 indicate that the polymorphism is probably damaging to protein function. Scores of 1.5–2.0 mean that it is possibly damaging, and scores of <1.5 signify that it is likely benign [17,18]. SIFT is a sequence homology–based tool that sorts intolerant from tolerant amino acid substitutions and predicts whether an amino acid substitution in a protein will have a phenotypic effect. SIFT is based on the premise that protein evolution is correlated with protein function. Positions important for function should be conserved in an alignment of the protein family, whereas unimportant positions should appear diverse in an alignment. Positions with normalized probabilities less than 0.05 are predicted to be deleterious and, those greater than or equal to 0.05 are predicted to be tolerated [19].

### Statistical analysis

Pearson χ^2^/Fisher’s exact test was applied to make a comparison between two groups (cases versus controls) and the p values less than 0.05 were considered as significant. All tests were done using SPSS software for windows (version 11.5; SPSS Inc., Chicago, IL).

## Results

### Clinical details

Table 1 details the clinical characteristics of 20 patients with PCG (mean age at onset, 0–3 years; 14 male and 6 female) who met the inclusion criteria. Patients with characteristics detailed in the exclusion criteria, e.g., history of blood transfusion, TORCH infection, and drug intake in the mother during pregnancy, were excluded from the study. Enrolled patients were observed for an average of more than three years in a glaucoma clinic caring for patients with relatively advanced disease.

**Table 1:** Details of all PCG patients.

Patient ID	Age/Sex	Corneal Diameter (mm) OS/OD and clarity at diagnosis	IOP (left/right eye; mmHg)	Buphthalmos	Haabs’ Striae	Last Cup/Disc ratio	Treatment
PCG 1	1Y/F	14/14.5: 14/14.5	17/17	OU	NO	L0.8:1 R-Almost total Cupping	MEDICAL AND 1X TRAB/TRAB OU
PCG2	5Y/M	13.5/13.5: 12/13.5	28/25	OU	NO	L-0.6:1 R-0.6:1	MEDICAL AND 2X TRAB/TRAB OU
PCG3	2.5Y/M	11/11: 14/14.5	41,261	OU	NO	L-0.7:1 R- Hazy Media	MEDICAL AND 2X TRAB/TRAB OU
PCG4	14 months/M	10.5/11: 13/14	18/30	OD	NO	L- 0.5:1 R 0.8:1	MEDICAL AND 1X TRAB OD
PCG5	2.6Y/M	14.5/15.5: 15.5/16	18/26	OU	NO	L-0.7:1 R- Almost Total Cupping	MEDICAL AND 1X TRAB/TRAB OU
PCG6	1Y/M	14/15: 14/15	41,131	OU	NO	L-0.2:1 R-0.2:1	MEDICAL AND 2X TRAB/TRAB OU
PCG7	6Y/F	14/16: 19/20	20/26	OD	NO	L-0.7:1 R- Not Visible	MEDICAL AND 2X TRAB/TRAB OU
PCG8	2Y/M	15/24.5: 14/14.5	24/22	OU	NO	L-0.5:1 R-0.7:1	MEDICAL AND 2X TRAB/TRAB OU
PCG9	1.6Y/M	14/14.5: 14/14	20/18	OU	NO	L- 0.5:1 R-0.5:1	MEDICAL AND 1XTRAB/TRAB OU
PCG10	6months/F	12/12.5: 15/15.5	22/31	OU	NO	L-0.6:1 R-Hazy Media	MEDICAL AND 1X TRAB/TRAB OU
PCG11	2Y/M	14/14: 14/14.5	26/30	OU	NO	L- 0.6:1 R-0.6:1	MEDICAL AND 2X TRAB/TRAB OU
PCG12	2 months/M	13/13.5: 13/13	26/20	OU	NO	Hazy Media	MEDICAL AND 1X TRAB/TRAB OU
PCG13	8Y/F	12/12: 14/14.5	22/34	OU	NO	L-O.8:1 R- Hazy Media	MEDICAL AND 2X TRAB/TRAB OU
PCG14	1.6Y/M	12/13: 12/13.5	26/32	OU	NO	L-0.8:1 R-Almost total cupping	MEDICAL AND 1XTRAB/TRAB OU
PCG15	6M/F	13/14: 14/14.5	20/20	OU	NO	L-0.8:1 R-0.7:1	MEDICAL AND 1XTRAB/TRAB OU
PCG16	5.7Y/M	13/13.5: 13/14	27/31	OU	OD	L-0.7:1 R- 0.6:1	MEDICAL AND 2X TRAB/TRAB OU
PCG17	4Y/M	14/14.5: 13/13.5	22/25	OU	NO	L-0.6:1 R-0.5:1	MEDICAL AND 2X TRAB/TRAB OU
PCG18	13 months/F	15/15: 13/13.5	18/16	OU	NO	L-0.7:1 R-0.7:1	MEDICAL AND 1XTRAB/TRAB OU
PCG19	9 months/M	13/13.5: 14/14.5	22/28	OU	NO	L-0.5:1 R-0.8:1	MEDICAL AND 1XTRAB/TRAB OU
PCG20	3Y/M	15/15.5: 11/11	35/16	OS	NO	L-Total cupping R- 0.4:1	MEDICAL AND 1XTRAB/TRAB OS

(The age of onset for all individuals was by birth. Abbreviations; OD-right eye; OS-left eye; OU-both eyes; X-times; Trab/Trab, combined trabeculotomy and trabeculectomy)

### Molecular analysis

MtDNA sequencing following whole genome (2 ribosomal RNA [rRNAs], 22 transfer RNA [tRNAs], and 13 encoding subunits) amplification of mitochondrial DNA revealed a total of 195 nucleotide variations (Appendix 1) in PCG patients and 58 in controls (Table 2). Of the 195 nucleotide variations in patients, 43 (22.05%) were nonsynonymous, 82 (42.05%) were synonymous changes, and 30 were in RNA genes. A total of 39/195 (20.00%) variations were observed in the D-loop (hypervariable region), 19/195 (9.74%) in different rRNAs, 11/195 (5.64%) in tRNAs, 66/195 (33.84%) in complex I, 17/195 (8.71%) in complex III, 27/195 (13.84%) in complex IV, and 15/195 (7.69%) in complex V. Of 58 variations in the controls, 14 were nonsynonymous changes. SIFT and PolyPhen analysis of all nonsynonymous changes from patients revealed two pathogenic changes in the NADH-ubiquinone oxidoreductase chain 2 (ND2) and cytochrome oxidase subunit III (COXIII) subunits (Appendix 1). MtDNA variations associated with other diseases were also found in this study (Appendix 2). In one of the patients, insertion of cytosine introduced a frameshift change (p.Ile104AsnfsX26) in the cytochrome b (CYB) subunit of the electron transport chain (ETC). In another patient, a variation (G8572A) in ATpase8 leads to the introduction of a stop codon or termination at amino acid position 69.

**Table 2:** Mitochondrial DNA variations in controls.

Nucleotide change	Locus	Codon change	Change in protein	Type of mutation	Polyphen/ SIFT score	Pathogenic (Yes/No)	Frequency of variation
3591 G>A	ND1	CTG>CTA	p.T95T	SYN	NA	NA	1/20
3915 G>A	ND1	GGG>GGA	p.G203G	SYN	NA	NA	1/20
3918 G>A	ND1	GAG>GAA	p.E204E	SYN	NA	NA	1/20
3933 A>G	ND1	TCA>TCG	p.S209S	SYN	NA	NA	1/20
*3970 C>T	ND1	CTA>TTA	p.L222L	SYN	NA	NA	2/20
3996 C>T	ND1	AAC>AAT	p.N230N	SYN	NA	NA	1/20
4029 C>A	ND1	ATC>ATA	p.I241M	NS	Benign/0.07	NA	1/20
*4852 T>A	ND2	CTG>CAG	p.L128Q	NS	1.951/0.00	Yes	2/20
*5186 A>T	ND2	TGA>TGT	p.W239C	NS	1.982/0.03	Yes	1/20
5348 C>T	ND2	TAC>TAT	p.Y293Y	SYN	NA	NA	1/20
5351 A>G	ND2	CTA>CTG	p.L294L	SYN	NA	NA	1/20
*10310 G>A	ND3	CTG>CTA	p.T84T	SYN	NA	NA	1/20
10609 T>C	ND4L	ATA>ACA	p.M47T	NS	Benign/0.23	No	1/20
*11467 A>G	ND4	TTA>TTG	p.L236L	SYN	NA	NA	1/20
*11914 G>A	ND4	ACG>ACA	p.T385T	SYN	NA	NA	1/20
*12007 G>A	ND4	TGG>TGA	p.W416W	SYN	NA	NA	1/20
12073 C>T	ND4	TTC>TTT	p.F438F	SYN	NA	NA	1/20
*12107 C>T	ND4	CTC>CTT	p.T449T	SYN	NA	NA	1/20
12133 C>T	ND4	TCC>TCT	p.S458S	SYN	NA	NA	1/20
*12372 G>A	ND5	CTG>CTA	p.T12T	SYN	NA	NA	1/20
12373 A>G	ND5	ACT>GCT	p.T13A	NS	Benign/0.00	No	1/20
12406 G>A	ND5	GTT>ATT	p.V24I	NS	0.299/0.72	No	1/20
12486 C>T	ND5	CCC>CCT	p.P50P	SYN	NA	NA	1/20
*12561 G>A	ND5	CAG>CAA	p.Q75Q	SYN	NA	NA	1/20
13204 G>A	ND5	GTC>ATC	p.V290I	NS	0.710/1.00	No	1/20
12477 T>C	ND5	AGT>AGC	p.S47S	SYN	NA	NA	1/20
12681 T>C	ND5	AAT>AAC	p.N115N	SYN	NA	NA	1/20
13806 C>T	ND5	GCC>GCT	p.A490A	SYN	NA	NA	1/20
14058 C>T	ND5	TCC>TCT	p.S574S	SYN	NA	NA	1/20
*14783 T>C	CYB	TTA>CTA	p.L13L	SYN	NA	NA	2/20
14872 C>T	CYB	ATC>ATT	p.I42I	SYN	NA	NA	1/20
15119 G>A	CYB	GCA>ACA	p.A125T	NS	0.760/0.00	No	1/20
15172 G>A	CYB	GGG>GGA	p.G142G	SYN	NA	NA	1/20
15217 G>A	CYB	GGG>GGA	p.G157G	SYN	NA	NA	1/20
15385 C>T	CYB	TCC>TCT	p.S213S	SYN	NA	NA	1/20
15431 G>A	CYB	GCC>ACC	p.A229T	NS	0.033/0.03	No	1/20
15484 A>G	CYB	TCA>TCG	p.S246S	SYN	NA	NA	1/20
15670 T>C	CYB	CAT>CAC	p.H308H	SYN	NA	NA	1/20
6032 G>A	CO1	CAG>CAA	p.Q43Q	SYN	NA	NA	1/20
6320 T>C	CO1	CCT>CCC	p.P139P	SYN	NA	NA	1/20
6734 G>A	CO1	ATG>ATA	p.M277M	SYN	NA	NA	1/20
7316 G>A	CO1	ATG>ATA	p.M471M	SYN	NA	NA	1/20
7738 T>C	CO2	ACT>ACC	p.T51T	SYN	NA	NA	1/20
7762 G>A	CO2	CAG>CAA	p.Q59Q	SYN	NA	NA	1/20
8143 T>C	CO2	GCT>GCC	p.A186A	SYN	NA	NA	1/20
*8251 G>A	CO2	GGG>GGA	p.G222G	SYN	NA	NA	1/20
8503 T>G	ATP8	AAT>AAG	p.N46K	NS	0.090/1.00	No	1/20
8584 G>A	ATP6	GCA>ACA	p.A20T	NS	0.362/0.19	No	1/20
8594 T>C	ATP6	ATC>ACC	p.I23T	NS	1.579/0.27	No	2/20
8650 C>T	ATP6	CTA>TTA	p.L42L	SYN	NA	NA	1/20
*8684C>T	ATP6	ACC>ATC	p.T53I	NS	0.219/1.00	No	1/20
8718 A>G	ATP6	AAA>AAG	p.K64K	SYN	NA	NA	1/20
8812 A>G	ATP6	ACC>GCC	p.T96A	NS	0.908/0.03	No	1/20
*8865G>A	ATP6	GTG>GTA	p.V113V	SYN	NA	NA	1/20
8886 G>A	ATP6	AAG>AAA	p.K120K	SYN	NA	NA	1/20

Abbrevations: *Mitochondrial variations found both in patients and controls, Reported- http://www.mitomap.org, SYN-synonymous, NS-Not synonymous, NA- Not applicable, ND1-NADH dehydrogenase subunit 1; ND2-NADH dehydrogenase subunit 2 ; ND3-NADH dehydrogenase subunit 3 ;ND4-NADH dehydrogenase subunit 4 ; ND5-NADH dehydrogenase subunit 5; CO1-cytochrome c oxidase I ; CO2-cytochrome c oxidase II; ATPase6-ATP synthase subunit a (F-ATPase protein 6) ; ATPase8-ATP synthase protein 8 ; CYB-cytochrome.

Haplogroup/phylogenetic analysis of mtDNA showed that primary congenital glaucoma patients belonged to three macrohaplogroups, specifically M (4), N (15), and L (1). Fifty percent of the patients belonged to the H2a2a lineage of the N-derived haplogroup (Appendix 2). Other haplogroups to which different patients belonged were H1a3 (1), M3c1a (1), L3* (1), U2b (1), U2 (1), M49c (1), U5b2a1a (1), M5c1 (1), and R31a1 (1).

## Discussion

In this study, we screened 20 PCG cases for mtDNA variations. Forty-three nonsynonymous mtDNA variations were found in PCG patients in different mitochondrial genes. The highest number of nucleotide variations were recorded in complex I (66), followed by complex IV (27), complex III (17), and then complex V (15). Complex I is responsible for the pumping of protons (H^+^) from the matrix to the intermembrane space in association with complex III and IV, and 33.84% of nucleotide variations identified in this study were in complex I. Although the mitochondrial ETC is very effective in the reduction of oxygen to water, there is a constant “leak” of electrons from the ETC to oxygen, which results in the formation of superoxide anions. Dismutation of superoxide anions produces hydrogen peroxide as a secondary product, which can be converted to a highly reactive hydroxyl radical that can readily oxidize proteins, lipids, carbohydrates, DNA, and RNA [20].

MtDNA mutates 10 times more frequently than nuclear DNA due to its proximity to the ETC and lack of histones and other protective proteins [21]. Mitochondria are susceptible to oxidative damage, as reactive oxygen species (ROS) damage mitochondrial enzymes directly and alter mitochondrial membrane permeability, which may lead to cell death [22,23]. Most studies suggest that the majority of intracellular ROS produced by nonphagocytic cells are derived from mitochondria [24]. Several human diseases have been associated with mtDNA mutations, indicating that dysfunction of the components of oxidative phosphorylation encoded by the mitochondrial genome can be deleterious [25,26]. Abnormalities in mtDNA have proven to be associated with Leber’s hereditary optic neuropathy (LHON) [27], POAG, PEG, PACG, and other spontaneous optic neuropathies [13,27,28], as well as male infertility [29] and premature ovarian failure [30].

There have been very few studies showing the role of various haplogroups in the pathogenesis of glaucoma as compared to LHON. In the case of LHON, a strong association has been found for 11778G>A and 14484T>C primary mutations, but not for 3460G>A. In this study, the possible association of the mitochondrial haplogroup with the pathogenesis of glaucoma was studied. We observed that 50% of the patients belong to the H2a2a lineage of the N-derived haplogroup, and no specific mutation was found to be associated with this haplogroup. Four patients belonged to the M haplogroup and one to the L haplogroup. No specific mutation was found to be associated with the M or L haplogroups.

Recent studies have shown that G4580A (p.M37I) in *ND2* and G10398A (p.A114T) in *ND3* are associated with an increase in production of ROS due to altered complex I function [31,32]. G4580A (p.M37I) was present in one patient and G10398A (p.A114T) in eight patients in this study. The frequency of G10398A associated with haplogroups I, J, and K was found to be higher in our study population. Nine patients (45.00%) had changes associated with elevated ROS production. It has been reported that alterations in mitochondrial complex I causes cytochrome c oxidase deficiency. Pathogenic mutations in ND genes have been reported in POAG, PACG, and PEG [13,27]. Cytochrome c oxidase (COX or complex IV), the terminal enzyme of the respiratory chain catalyzes the reduction of molecular oxygen by reduced cytochrome c. This complex is composed of 13 subunits. Twenty-nine variations (14.14%) identified in this study were present in complex IV, of which seven were nonsynonymous. Human diseases associated with COX mutations include POAG, PACG, PEG, and Leigh syndrome [9,13].

In the current study, 7.31% mtDNA variations (15/205) were observed in complex V (*ATPase6* and *ATPase8*). Mutations in *ATPase6* have been reported in POAG, PACG, PEG, neuropathy, ataxia, retinitis pigmentosa and mitochondrial DNA-associated Leigh syndrome patients [9,13,33,34]. Mitochondrial variations in *ATPase6* and *ATPase8* have been reported in spinocerebellar ataxias [35]. The A12308G variation in tRNA leu gene is also associated with increased ROS production [32] and this variation was detected in three patients in our study. However, the frequency of the A12308G variation and non-synonymous variations in *ATPase8* was not high, while those of nonsynonymous variations in *ATPase*6 and others (16s RNA, tRNA) were greater.

Nonsynonymous mitochondrial variations can adversely affect oxidative phosphorylation, which may result in decreased mitochondrial respiration [36]. Thus, mtDNA variations may lead to mitochondrial dysfunction, resulting in lower ATP levels that may also impair the growth, development, and differentiation of the trabecular meshwork (TM), and can result in trabecular dysgenesis, a characteristic feature of PCG. Trabecular dysgenesis leads to impairment in aqueous drainage, hence causing elevation in IOP. ROS levels may increase to supraphysiological levels in TM endothelial cells, and due to low ATP levels, these cells are unable to eliminate the reactive oxygen intermediates. The mechanisms by which mitochondrial abnormalities may place the optic nerve at risk remain uncertain. Any malfunction of the mitochondrial ETC results in excessive generation of free radicals and low ATP production. In our study, we identified a higher number of mtDNA nucleotide variations in complex I as compared to other complexes of oxidative phosphorylations (OXPHOS). None of the PCG cases had primary LHON mutations (3460G>A, 11778G>A, 14484T>C) in the current study.

It has already been reported that OS leads to oxidative damage to cellular macromolecules such as mitochondrial and nuclear DNA, proteins, and lipids, along with energy depletion and a local dysregulation of calcium homeostasis, resulting in neuronal degeneration [37]. OS is the underlying etiology in several ocular diseases [37-40] and plays an essential role in early retinal ischemic injury [41] and the pathogenesis of glaucoma [42,43]. Glaucomatous eyes have a significant increase in OS and decreased antioxidant activity [43]. Seppet et al. [44] reported that OS is a critical factor in injury to the anterior segment of the eye. OS has also been reported to induce degenerative changes in the human TM that favor increased IOP [45]. The pathogenic role of ROS in glaucoma is supported by various experimental findings, including the following: (a) Resistance to aqueous humor outflow is increased by hydrogen peroxide by inducing TM degeneration, and (b) IOP increase and the severity of visual loss in glaucoma patients parallel the amount of oxidative DNA damage affecting TM. OS thus affects both the TM and RGCs, and may be involved in the neuronal cell death affecting the optic nerve in glaucoma.

Thus, the structure and function of mitochondria are critical determinants of endothelial cell function and neuronal health. It has been established that pathogenic mitochondrial mutations can cause mitochondrial dysfunction and enhance OS, which in turn lead to apoptosis in affected tissue [37] and accumulation of large number of synonymous variations may reduce the efficiency of translations by codon bias. There are several studies which point to mitochondrial dysfunction in glaucoma and RGC death [37,46]. One hypothesis suggests that progressive optic nerve damage in POAG is the result of optic nerve fiber apoptosis [47]. Mitochondria-induced apoptosis, which may be a mechanism of injury in experimental glaucoma [47] and other optic neuropathies [37], may also be a pathological factor in PCG. A study by [9] reported mitochondrial dysfunction-associated OS as a risk factor for glaucoma. MtDNA alterations result in reduced mitochondrial respiration and OS. Thus reduced ATP levels secondary to mitochondrial damage may impair development and differentiation of TM. Endothelial cells are also damaged due to supraphysiological ROS levels. These findings suggest that elevation of IOP is related to oxidative degenerative processes affecting the TM specifically endothelial cells. Much evidence indicates that in this region ROS play a fundamental pathogenic role by reducing local antioxidant activities inducing outflow resistance. TM is neural crest in origin [48] and developing TM is deficient in antioxidant enzymes and more susceptible to OS induced DNA damage [49]. OS, early in development and/or throughout life could precipitate both metabolic and anatomic sequelae that cause trabecular dysgenesis and ultimately optic nerve damage in PCG.

The precise relationship between among elevated IOP, glaucomatous optic nerve (ON) damage, and RGC death are poorly understood. Growing evidence indicates that mitochondrial structural and functional dynamics play an important role in cell and tissue physiology. Elevated IOP in glaucoma induces reduction of COX activity, mitochondrial fission, mitochondrial matrix swelling, and cristae depletion, and triggers release of optic nerve atrophy type-I, as well as inducing subsequent apoptotic cell death in differentiated RGC-5 cells [50,51]. Similar findings were also confirmed in a mouse model [50].

In summary, the frequency of mtDNA sequence variations in PCG was significantly higher as compared to controls. Nonsynonymous mtDNA alterations may lead to mitochondrial dysfunction which leads to reduced mitochondrial respiration, OS, damage to mtDNA, altered mitochondrial morphology, alterations in mitochondrial fission and fusion, and ultimately the cell’s demise. This study describes mtDNA sequence variations in PCG cases of a north Indian ethnicity. Knowledge of mtDNA mutations and/or mitochondrial dysfunction in PCG may lead to a better understanding of glaucoma the pathophysiology of glaucoma as shown earlier [52]. Novel approaches are now available for studying mitochondrial disease in the eye, and a novel in vitro treatment has already been devised for the metabolic defect of at least one mtDNA mutation in LHON [53]. PCG cases with mtDNA variations and consequent OS may benefit by early diagnosis and prompt management with antioxidant therapy.

### Conclusion

A total of 195 mtDNA variations were identified in this study. MtDNA variations adversely affect the respiratory chain; impair the OXPHOS pathway, resulting in low ATP production; and impair the growth, development, and differentiation of TM. Mitochondrial DNA variations also lead to increased ROS production, as well as oxidative injury to TM and RGCs. Thus, early diagnosis of mitochondrial DNA variations and prompt antioxidant administration may delay OS-induced injury to the TM and RGCs, thereby improving visual prognosis. Although several mutations were found at a higher frequency among our population, there is a need to complement this study through functional studies and to analyze large samples in different populations of different haplogroups, as penetrance varies among haplogroups.

## Appendix 1. Mitochondrial DNA variations in patients

Abbrevations: SYN-synonymous, NS-Not synonymous, NA-Not applicable, ND1-NADH dehydrogenase subunit 1; ND2-NADH dehydrogenase subunit 2 ; ND3-NADH dehydrogenase subunit 3 ;ND4-NADH dehydrogenase subunit 4 ; ND5-NADH dehydrogenase subunit 5; CO1-cytochrome c oxidase I ; CO2-cytochrome c oxidase II; ATPase6-ATP synthase subunit a (F-ATPase protein 6) ; ATPase8-ATP synthase protein 8 ; CYB-cytochrome. To access the data, click or select the words “Appendix 1.” This will initiate the download of a compressed (pdf) archive that contains the file.

## Appendix 2. Disease associated mtDNA nucleotide changes observed in the PCG patients, their significance and haplogroups.

To access the data, click or select the words “Appendix 2.” This will initiate the download of a compressed (pdf) archive that contains the file. (rCRS – revised Cambridge Reference Sequence)
